# Biomarkers in Psychiatric Disorders

**DOI:** 10.1017/S0963180122000056

**Published:** 2022-10

**Authors:** Walter Glannon

**Affiliations:** Department of Philosophy, University of Calgary, Calgary, Alberta T2N 1N4, Canada

**Keywords:** biomarkers, diagnosis, major depressive disorder, prediction, prevention, psychiatry, schizophrenia, treatment

## Abstract

Central and peripheral biomarkers can be used to diagnose, treat, and potentially prevent major psychiatric disorders. But there is uncertainty about the role of these biological signatures in neural pathophysiology, and their clinical significance has yet to be firmly established. Psychomotor, cognitive, affective, and volitional impairment in these disorders results from the interaction between neural, immune, endocrine, and enteric systems, which in turn are influenced by a person’s interaction with the environment. Biomarkers may be a critical component of this process. The identification and interpretation of biomarkers also raise ethical and social questions. This article analyzes and discusses these aspects of biomarkers and how advances in biomarker research could contribute to personalized psychiatry that could prevent or mitigate the effects of these disorders.

## Introduction

Major depression, bipolar disorder, schizophrenia, generalized anxiety, and other major psychiatric disorders are characterized by varying degrees of psychomotor, cognitive, affective, and volitional impairment. They are not simply disorders of the mind or brain but multisystem disorders that alter mental content and disable mental and motor capacities. Genome-wide association studies have implicated multiple genes in these disorders.^1^ Yet whether genes contribute to pathophysiology in the brain is influenced by epigenetic factors associated with the environment and chronic psychosocial stress. Neuro-endocrine interaction in response to stress can cause dysregulation in the hypothalamic–pituitary–adrenal axis. This can induce pro-inflammatory activation leading to depression and anxiety.^2^ Neuro-immune interaction can trigger high cytokine levels and excess microglia-mediated synaptic pruning in adolescence. This process has been associated with the onset of schizophrenia.^3^ There is also evidence of dysfunctional connectivity between gut microbiota and the central nervous system (CNS) in anxiety and depression.^4^ These examples illustrate that psychiatric disorders are complex disorders involving dysregulated interaction between mind, brain, body, and environment.

Because of variability in these interactions among people, there is considerable heterogeneity in the nature and extent of psychiatric disorders. There is also a high rate of comorbidity and overlap in neural dysfunction among neurological and psychiatric disorders. Movement disorders like Parkinson’s disease and cognitive and mood disorders like major depression and schizophrenia involve similar dysregulated connections in motor, associative and limbic circuits of the basal ganglia and cerebral cortex.^5^ This may involve overlap in motor symptoms in conditions such as catatonia. Yet treatments for these disorders typically target different neurotransmitters. All these factors pose diagnostic and therapeutic challenges. They are medically and morally significant because psychiatric disorders constitute a high percentage of the global burden of disease.^6^

Biomarkers are one area of research in biological psychiatry that could meet and possibly overcome these challenges.^7^ Central biomarkers in the brain and peripheral biomarkers such as proteins in blood, plasma, and tissue are biological signatures that have led to a better understanding of the origin and pathophysiology of psychiatric disorders. Identifying biomarkers not just in the CNS but also in other bodily systems with which it interacts may lead to more accurate diagnosis and more effective treatments targeting dysfunctional pathways in these disorders. It also has the potential to predict and prevent them in a patient-specific way. This would be one form of personalized psychiatry. But it would depend on how the interaction of biological, psychological, and social factors influences the effects of biomarkers on the brain and mind. Biomarkers raise ethical and social questions that must be addressed together with their therapeutic and preventive potential.

## Biomarkers in Diagnosis and Treatment

Structural and functional neuroimaging can identify central biomarkers in psychiatric disorders. Diffusion tensor imaging (DTI) tractography showing abnormalities in deep white matter tracts in Bipolar I disorder can distinguish it from abnormalities in these tracts and neural circuits implicated in major depression.^8^ These different neural signatures can enable diagnostic clarification of these disorders, with important implications for therapy. Antidepressants targeting serotonin receptors can control symptoms of major depression. But they can precipitate mania and mood cycling in bipolar disorder, for which lithium is the most effective therapy.^9^ In addition, imaging showing hypermetabolism in the amygdala and hypometabolism in the ventral tegmental area could help to diagnostically distinguish anxiety from depression.^10^ These disorders often overlap but may not respond to the same treatment. Precise identification of distinct biomarkers could enable psychiatrists to tailor therapy to individuals affected by them.

Imaging of biomarkers can also determine which pharmacological treatments for major depressive disorder (MDD) are effective or ineffective for specific individuals who already have been diagnosed.^11^ They could also be used to measure and monitor responses to cognitive behavioral therapy (CBT), electroconvulsive therapy (ECT), transcranial magnetic stimulation (TMS), or deep brain stimulation (DBS), as well as how these responses compare with each other. This could ameliorate the problem of treatment resistance in depression. Eleven years ago, Paul Holtzheimer and Helen Mayberg reported that “approximately 10–20% of depressed patients may show little or no improvement, despite multiple, often aggressive, treatments.”^12^ This number is likely higher today. Patients who fail to respond to antidepressant medication may respond to different forms of neuromodulation. Biomarkers specific to each patient’s brain could determine which of these interventions would be more likely to modulate neural and mental function and control depression. They could also be used to guide interventions to control other disorders.

In a study involving patients with MDD, hypometabolism in the insula displayed by PET was associated with a positive response to CBT in terms of symptom reduction and a poor response to the SSRI escitalopram. Hypermetabolism in the insula was associated with a positive response to the drug and a poor response to CBT. The study showed that a treatment-specific biomarker could guide therapy selection for patients with MDD.^13^ More recent studies of the neurobiological basis of depression have shown dysregulated connectivity and function in cortical and limbic regions due to alterations in excitatory glutamate neurons and inhibitory GABA interneurons.^14^ These biomarkers may indicate that pharmacological agents such as the NMDA receptor antagonist ketamine can target and modulate these neurotransmitters more effectively than standard antidepressants that target monoamine neurotransmitters.^15^ Such novel treatments can improve depressive symptoms and avoid adverse effects.

Imaging of hyperactivity or hypoactivity in cortical–limbic circuits may distinguish the subtypes of schizophrenia corresponding to positive symptoms such as hallucinations and delusions and negative symptoms such as flat affect, anhedonia, and avolition.^16^ One imaging study of schizophrenia patients with first-episode psychosis showed cortical–striatal dysconnectivity. This led researchers to hypothesize that increased functional connectivity between the striatum and prefrontal and limbic regions may be a biomarker for symptom improvement from antipsychotic medication.^17^ Central biomarkers showing abnormalities correlating with negative symptoms could indicate different treatments. In addition to benefiting patients by relieving debilitating symptoms of psychiatric disorders, these and other biomarker-guided therapies can reduce harm by avoiding adverse neurophysiological and psychological effects.

## Predictive Biomarkers: Potential and Limitations

Preclinical identification of biomarkers correlating with dysregulated neural, neuro-immune, and neuro-endocrine interaction could indicate early therapeutic or preventive interventions. “More refined use of biomarkers might be beneficial, for example, if a biomarker could predict the presence of an early disorder that is not yet clinically evident but would show improved outcome with early treatment.”^18^ This could mitigate symptoms of psychiatric disorders and possibly prevent full-blown development. These signatures might be detected as part of screening or testing of individuals deemed at risk based on genetic inheritance. Imaging showing changes in chromatin structure at particular genomic loci in limbic regions may indicate changes in gene expression in these regions that contribute to depression.^19^ These are epigenetic changes induced by stressful environmental stimuli. They may have predictive value for individuals at risk of depression because “such stress-induced epigenetic modifications also occur early in life and help determine an individual’s lifetime vulnerability or resistance to subsequent stressful events.”^20^ Altering one’s environment would be a nonpharmacological intervention that could control the effects of this process, though this may depend on income, social mobility, and other environmental factors that influence epigenetic processes.

Hyperactive synaptic pruning in the adolescent brain may be a biomarker for schizophrenia. This is one hypothesis for the onset of this disorder and why many psychiatric disorders begin in adolescence. Pathogenesis is often associated with neuroinflammation from microglial activation and thus an example of dysregulated neuro-immune interaction. PET imaging has detected increased microglial activity in subjects at high risk of psychosis.^21^ MRI or DTI tractography can also detect this and other forms of neuro-immune dysregulation. One possible early intervention would be the antibiotic minocycline to modulate the rate of synaptic pruning.^22^ Whether this intervention was safe and effective could only be confirmed by controlled clinical trials. Among the issues these trials would have to clarify is whether minocycline or any other agent administered through the blood–brain barrier would be able to induce or maintain an optimal level of synaptic pruning for normal brain development.

Some biomarkers detected preclinically in individuals at risk could identify targets for early intervention in MDD. One hypothesis for the incidence of treatment resistance in this disorder is that allowing abnormalities in cortical–limbic connectivity to develop may cause permanent changes in this connectivity. This could make these abnormalities and the disordered mental states associated with them intractable to modulation through medication or neurostimulation. Early identification of these biomarkers and intervention could prevent these changes and control or even stop disease progression. One example of what could be a predictive biomarker for MDD involving gene–environment interaction is the combination of the CACNA1C rs1006737 polymorphism and life-threatening events that influence its expression. These events can negatively influence transcription factors in brain regions mediating cognitive and affective processing.^23^ New pharmacological treatments that could modulate these factors could be part of a preventive strategy. But this hypothesis would have to be tested and confirmed by further research.

Although many central and peripheral biomarkers associated with psychiatric disorders have been identified, their diagnostic, therapeutic, and especially predictive value are fraught with uncertainty. A biomarker as such is not indicative of neuropathology. Just because it is correlated with a disease does not entail that it will cause that disease. It is not clear whether abnormalities in neural and other systems associated with psychiatric disorders are already present before symptoms appear, or whether these abnormalities occur as a consequence of psychotic or depressive episodes. This issue is especially pertinent to depression, anxiety, and obsessive–compulsive disorder. Longitudinal studies of individuals with these disorders may answer this question. Imaging or blood samples detecting biomarkers at a specific time provide limited information about the probability of developing one of these disorders. Even if brain abnormalities associated with these disorders are present at an early age, they may not manifest in disease. Administering pharmacological agents based on biomarkers could prevent psychiatric disorders from developing. But it could also unnecessarily cause neurophysiological and psychological sequelae if the disease did not develop. Because the connection between biomarkers and these disorders is probabilistic rather than deterministic, the risk of allowing them to develop without early intervention must be weighed against the risk of sequelae from intervention. The complex relations between genetic, epigenetic, neural, immune, endocrine, enteric, and environmental factors account for the variability in the incidence of psychiatric disorders and heterogeneity in symptoms among affected individuals over the course of their lives. A biomarker is just one component of the pathophysiology of these disorders.

A biomarker such as dysconnectivity between frontal, parietal, and striatal pathways may be a precursor of schizophrenia. This could be detected by neuroimaging in an adolescent deemed at risk because of an affected parent. Correlations between this and other neural signatures and prodromal signs may indicate early antipsychotic therapy. The prodrome is the phase of a psychiatric disorder when nonspecific changes in thought and behavior precede overt symptoms. Neural dysconnectivity could be combined with prodromal signs such as impaired sensory gating, attention, and working memory to indicate administering an antipsychotic drug before a first-episode psychosis.

Early intervention may stop or pre-empt pathogenesis and the development of a disorder. Yet the potential adverse effects of antipsychotic or other psychotropic medications taken before a definitive diagnosis has been made raise questions about biomarker-based treatment during the prodrome. Rather than improving cognitive and affective functions, these medications may impair them. Specifically, they may impair attention and working memory.^24^ Substantial differences in cognitive, affective, and psychomotor functions among individuals at risk of schizophrenia complicate estimating outcomes of preventive psychopharmacology. Regarding positive symptoms in schizophrenia, “there is considerable heterogeneity within clinical high-risk samples because studies have consistently observed that most high-risk individuals do not go on to develop clinical psychosis, and a substantial minority may even recover symptomatically and functionally. Future research should focus on understanding the neurocognitive and psychosocial factors that characterize nonconverters, as well as those who recover.”^25^ This underscores the questionable predictive value of biomarkers in schizophrenia and other psychiatric disorders when considered independently of other factors. In some cases, early biomarker-guided interventions may be more harmful than beneficial. Further research is necessary to clarify disease risk and whether pharmacological treatment is or is not indicated. Intervening in cases where disease and symptoms did not eventually appear could adversely affect normal brain development.

This is a particular concern regarding children and adolescents. In some cases, individuals as young as 11 or 12 years of age may have nonspecific psychotic or other positive and negative symptoms of schizophrenia. “Clearly this presents the possibility of early intervention in late childhood. However, only some individuals with psychotic-like experiences go on to develop schizophrenia, and this highlights the need to develop effective biomarkers to identify those individuals at risk who will actually show schizophrenia.”^26^ Again, these biomarkers provide a probabilistic rather than deterministic framework in predicting whether an individual with them will develop the disorder. This depends on how neuro-immune and neuro-endocrine interaction, as well as the individual’s adaptability to the physical and social milieu, influence neuroanatomical and neurophysiological abnormalities implicated in schizophrenia and other disorders. Whether or to what extent an individual will develop a disorder depends on the degree of consonance or dissonance between their brain and the environment in which they live.^27^ The heterogeneity of these disorders is due at least partly to differences in these environments and adaptability to them. While the examples I have presented illustrate how biomarkers can contribute to more accurate diagnosis and treatment, they also illustrate that they have limited predictive value on their own.^28^

Psychiatric disorders are moderate to highly heritable. Approximately 60–65% of the risk associated with schizophrenia is attributable to genes. In contrast, approximately 30–40% of the risk for MDD is attributable to them.^29^ The remaining risk is associated with environmental factors. There may be hundreds of genes associated with these disorders. Identifying particular genes or gene variants will not establish a causal connection between them and disease. Researchers must collect genetic data from large cohorts of research subjects for it to be statistically significant. Even with the data, “the degree to which genetic variation is unique to individual disorders or shared across disorders is unclear.”^30^ More unclear is whether a particular person with a genetic susceptibility to a particular disorder will develop it. “Big Data” and genome-wide association studies cannot resolve these issues. Statistical analysis alone cannot explain why some people at risk develop a disorder while others do not. Having a first-degree relative with schizophrenia, for example, may entail a higher probability of developing this disorder. But it does not determine that one will in fact develop it. The difficulty in clarifying correlations between genes and disease is not just the number of genes identified but also how they are expressed or inhibited by epigenetic factors. Psychiatric genetic research has resulted in a better understanding of the role of genes in these disorders, particularly schizophrenia and depression.^31^ But they are just one feature of the underlying biology.

Inferences from biomarkers to a specific disorder “must be shown to be robust and valuable at the individual level.”^32^ Biomarkers could be clinically relevant only if they had strong diagnostic, therapeutic, and predictive value for individuals. The main challenge in achieving this goal is that probabilistic risk assessment about developing a psychiatric disorder is based on genetic analysis and neuroimaging involving group studies. Statistical analysis alone cannot explain why a disorder affects some people at risk and not others.^33^ Researchers conducting these studies cannot draw direct inferences from information about populations or groups to information about individuals within these groups. This applies not only to prediction but also to treatment, given variability in how individuals with schizophrenia, depression, or other major psychiatric disorders respond to medication and other interventions. It also applies to the extent to which they regain and maintain cognitive affective, and motor functions and re-engage with the world from different therapies. An understanding of how biomarkers interact with other bodily systems and the environment in a patient-specific way is necessary for their identification to be effective in therapy and prevention.

There are cases in which early detection of biomarkers could be critical in preventing harm. Brain biomarkers could identify individuals at risk of suicide from severe depression. Suicide is the greatest harm from this and other psychiatric disorders. The incidence of treatment resistance in major depression may be due to failure to intervene at an early stage of the disease to modulate dysfunctional neural circuits implicated in it. This could accelerate dysfunction, pathogenesis, and possible neurodegeneration, making the disorder resistant not only to antidepressants but also to ECT, TMS, and DBS. Treatment resistance, especially when it is prolonged, is one factor leading to suicide among these patients. Neuroimaging studies across different psychiatric disorders have shown structural, functional, and molecular alterations correlating with suicidal thought and behavior. These studies suggest that impairments in medial and lateral regions of the ventromedial prefrontal cortex and the dorsal prefrontal cortex, and connections between these and other brain regions, can induce negative mental states and lead to suicidal ideation and suicide attempts.^34^ Early intervention targeting dysfunction in these neural networks at the first sign of maladaptive thought and behavior could modulate these networks and prevent suicide in severe depression. A recent study showed that ketamine could rapidly decrease suicidal ideation in participants with bipolar disorder.^35^ The risk of harm from delayed intervention can be greater than the risk of harm from early intervention and potential adverse effects from psychotropic drugs. These considerations support the claim that intervening in the brain based on preclinical evidence of particular biomarkers can be justified. Indeed, given the magnitude of the potential harm from suicidal thought and behavior, early therapy for individuals with biomarkers indicating a high risk of taking their own lives would be obligatory.

## Social Issues

Screening and testing adolescents at risk of MDD based on genetic and other biomarkers can be conducted in primary care settings. This can be supplemented by physicians engaging with patients in discussing their overall well-being.^36^ For those at high risk, appropriate interventions could prevent this disorder or mitigate its effects. In 2012, it was reported that “more than 30 randomized trials have demonstrated that preventive interventions can reduce the incidence of new episodes of MDD by about 25% and by as much as 50% when preventive interventions are offered in stepped-care format. Methods with proven effectiveness involve educational, psychotherapeutic, pharmacological, lifestyle and nutritional interventions.”^37^ But screening, testing, and interventions based on them would be costly and not sustainable for many health care systems. If they were sustainable, then it would be in higher-income countries. People at risk in lower-income countries would not be screened or tested. This would result in unequal access to therapeutic or preventive interventions, depending on where one lived, which would be beyond most people’s control. It would result in an unfair distribution of the burden of psychiatric disorders between the socioeconomically better and worse off when all had the same medical need. Those who had no access to preventive programs or therapies could develop a disorder and suffer from untreated symptoms that could affect them for the balance of their lives. The issue is not neurobiology but the need for equal provision and effective delivery of mental health services in communities across the globe.

There are other potential social implications of identifying and interpreting biomarkers in children and adolescents. “Biomarker information might reshape the beliefs, practices and decision-making of the people in a child’s environment, including parents, teachers and health providers.”^38^ This could influence how they interpret the child’s behavior and adversely affect their interactions with the child. The belief that a child might be predisposed to a psychiatric disorder could lead their parents, teachers, and others to limit opportunities for them and their ability to develop autonomous agency, interests, and undertake and complete life plans. It could limit the child’s right to an open future.^39^

Depending on how they were interpreted, biomarkers associated with possible future psychiatric disorders might also contribute to or exacerbate the stigma associated with them. Phenotypically normal people might experience discrimination based on a known biomarker without any sign of disease or cognitive and affective impairment. Private health insurance companies with access to biomarker information might unfairly deny insurance to individuals who may or may not develop a costly medical condition. Companies that provide health insurance to their employees and have this information could deny employment to individuals for the same reason. More disturbing would be the use of this information in children to make questionable inferences about future antisocial or criminal behavior. Some interventions might be justifiable when there was evidence of this behavior at the time a biomarker was identified. But psychotropic medication based on biomarker risk alone would be difficult to justify because it could impair natural neural development. The risk of harmful effects on the child’s brain that could persist over their lifespan could outweigh the risk of harmful behavior from not altering their brain. These considerations underscore the ethical and legal obligations of researchers studying connections between neural biomarkers and future behavior among children and adolescents to protect them and their families from different types of harm.^40^ The US Genetic Information Nondiscrimination Act of 2008 prohibits the discriminatory use of genetic information in health insurance and employment. Similar legislation may be necessary to protect children and adolescents from misuse of information about their brains.

## Conclusion

Many psychiatrists endorse the biopsychosocial model to explain the pathophysiology of psychiatric disorders.^41^ They also use it to guide interventions to control and mitigate their harmful effects on affected individuals. In schizophrenia, the “psycho” and “social” components are not limited to individual or group psychotherapy but can be part of a comprehensive four-pronged early intervention after first-episode psychosis. In addition to antipsychotic medication, this includes psychotherapy, social and professional support to continue at work or school, and education of family members to improve their understanding of the disease.^42^ Other examples of a holistic approach to the psychosocial aspect of therapy are improved patient-centered care and architectural design of psychiatric treatment centers such as the Menninger Clinic in Houston (https://www.menningerclinic.org) and the Taube Pavilion in Mountain View, California (https://www.elcaminohealth.org/health-behavioral-health-services-taube-pavilion).

Central and peripheral biomarkers are only one component of the biopsychosocial model. But they are a critical component that could yield greater insight into differences between healthy and diseased brains and minds. Advances in research that not only identifies biomarkers but also shows how they interact with other biological, psychological, and social factors can lead to a better understanding of the pathophysiology of psychiatric disorders. They could also lead to more accurate diagnosis, more effective treatment, and early interventions that could prevent them from developing into intractable diseases.

Despite the identification of an increasing number of biomarkers associated with major psychiatric disorders, thus far they have not resulted in significant clinical improvement in the psychiatric patient population. Translating findings of neuroanatomical and neurophysiological signatures from neuroimaging or blood samples into psychiatric practice requires a better understating of how other biological factors and an individual’s adaptability to the environment influence their expression.^43^ It is not clear that advances in biomarker research could overcome all the challenges of variability and heterogeneity in whether or to what extent individuals develop and are affected by psychiatric disorders. Larger neuroimaging data sets will not answer these questions. Screening and testing children and adolescents deemed at risk of having one of these disorders from biomarkers would advance understanding of the relation between biomarkers and disease. As noted, though, these interventions would be expensive and not sustainable for most health care systems. They could also result in unequal access to them. In addition, they could lead to bias and discrimination against people with these biological signatures based on questionable inferences from these signatures to thought and behavior. These are some of the methodological and logistical challenges in achieving the goal of personalized psychiatry. Nevertheless, combining biomarker research and longitudinal studies of affected individuals could clarify their causal role in schizophrenia, major depression, and other disorders. This could go some way toward reducing the global burden of disease and improve the quality of life for the hundreds of millions of individuals who suffer from them.

## Notes

1.

McIntosh A , Sullivan P , Lewis C. Uncovering the genetic architecture of major depression. Neuron 2019;102:91–103 3094683010.1016/j.neuron.2019.03.022PMC6482287.

2.

Engert V , Grant J , Strauss B. Psychosocial factors in disease and treatment—A call for the biopsychosocial model. JAMA Psychiatry 2020;77:996–73249208210.1001/jamapsychiatry.2020.0364.

3.

Momtazmanesh S , Zare-Shahabadi A , Rezaei N. Cytokine alterations in schizophrenia: An updated review. Frontiers in Psychiatry 2019;10:892 3190864710.3389/fpsyt.2019.00892PMC6915198. doi:10.3389/fpsyt.2019.00892; Cuenod M , Steullet P , Cabungcal J-H , Dwir D , Khasimallah I , Klauser P , et al. Caught in vicious circles: A perspective on dynamic feed-forward loops driving oxidative stress in schizophrenia. Molecular Psychiatry 2021;26:1374 10.1038/s41380-021-01374-wPMC912681134759358. doi:10.1038/s41380-021-01374-w.

4.

Caso J , MacDowell K , Gonzalez-Pinto A , Garcia S , de Diego-Adelino J , Carcellar-Sindreu M , et al. Gut microbiota, innate immune pathways, and inflammatory control mechanisms in patients with major depressive disorder. Translational Psychiatry 2021;11:645. doi:10.1038.s41398-021-01755-33493404110.1038/s41398-021-01755-3PMC8692500.

5.

Perju-Dumbrava L , Kempster P. Movement disorders in psychiatric patients. BMJ Neurology Open 2020;2:e00005710.1136/bmjno-2020-000057PMC787172433681793. doi:10.1136/bmjno-2020-00057.

6.

Vigo D , Thornicroft G , Atun R. Estimating the true global burden of mental illness. The Lancet Psychiatry 2016;3:171–82685133010.1016/S2215-0366(15)00505-2.

7.

Garcia-Gutierrez M , Manzanares J , Navarrete F. The search for biomarkers in psychiatry. Frontiers in Psychiatry 2021;12:72041110.3389/fpsyt.2021.720411PMC831094434322046. doi:10.3389/fpsyt.2021.720411.

8.

Sarrazin S , Poupon C , Linke J , Wessa M , Phillips M , Delevest M , et al. A multicenter tractography study of deep white matter tracts in bipolar 1 disorder: Psychotic features and dysconnectivity. JAMA Psychiatry 2014;71:388–962452219710.1001/jamapsychiatry.2013.4513.

9.

Strakowski S. Bipolar Disorder. New York: Oxford University Press; 2014:59 .

10.

Oathes D , Patenaude B , Schatzberg A , Etkin A. Neurobiological signatures of anxiety and depression in resting-state fMRI. Biological Psychiatry 2015;77:385–932544416210.1016/j.biopsych.2014.08.006PMC4297561.

11.

Strawbridge R , Young A , Cleare A. Biomarkers for depression: Recent insights, current challenges and future prospects. Neuropsychiatric Disease and Treatment 2017;13:1245–622854675010.2147/NDT.S114542PMC5436791.

12.

Holtzheimer P , Mayberg H. Stuck in a rut: Rethinking depression and its treatment. Trends in Neurosciences 2011;34:1–9, at 22106782410.1016/j.tins.2010.10.004PMC3014414.

13.

McGrath C , Kelley M , Holtzheimer P , Dunlop B , Craighead W , Franco A , et al. Toward a neuroimaging treatment selection biomarker for major depressive disorder. JAMA Psychiatry 2013;70:821–9.2376039310.1001/jamapsychiatry.2013.143PMC4413467

14.

Duman R , Sanacora G , Krystal J. Altered connectivity in depression: GABA and glutamate neurotransmitter deficits and reversal by novel treatments. Neuron 2019;102:75–90 3094682810.1016/j.neuron.2019.03.013PMC6450409.

15.

Sanacora G , Frye M , McDonald W , Mathew S , Turner M , Schatzberg A , et al. A consensus statement on the use of ketamine in the treatment of mood disorders. JAMA Psychiatry 2017;74:399–405 2824907610.1001/jamapsychiatry.2017.0080.

16.

Castle D , Buckley P. Schizophrenia . 2nd ed. New York: Oxford University Press; 2015:13–15 .

17.

Sarpal D , Robinson D , Lencz T , Argyelan M , Toshikazu I , Karlsgodt K , et al. Antipsychotic treatment and functional connectivity of the striatum in first-episode schizophrenia. JAMA Psychiatry 2015;72:5–13 2537284610.1001/jamapsychiatry.2014.1734PMC4286512.

18.

Boksa P. A way forward for research on biomarkers for psychiatric disorders. Journal of Psychiatry and Neuroscience 2013;38:75–7, at 75 2342205210.1503/jpn.130018PMC3581594.

19.

Nestler E. Epigenetic mechanisms of depression. JAMA Psychiatry 2014;71:454–62449992710.1001/jamapsychiatry.2013.4291PMC4057796.

20. See note 19, Nestler 2014, at 454.

21.

Bloomfield P , Selvaraj S , Veronese M , Rizzo G , Bertoldo A , Owen D , et al. Microglial activity in people at ultra high risk of psychosis and in schizophrenia: An [(11) C] PBR 28 PET imaging study. American Journal of Psychiatry 2016;173:44–52 2647262810.1176/appi.ajp.2015.14101358PMC4821370.

22.

Inta D , Lang U , Borgwardt S , Meyer-Lindenberg A , Gass P. Micoglia activation and schizophrenia: Lessons from the effects of minocycline on postnatal neurogenesis, neuronal survival and synaptic pruning. Schizophrenia Bulletin 2017;43:493–6.2735278210.1093/schbul/sbw088PMC5464012

23.

Zhao M , Yang J , Qiu X , Yang X , Qiao Z , Song X , et al. CACNA1C rs1006737, threatening life events, and gene–environment interactions predictive of major depressive disorder. Frontiers in Psychiatry 2020;10:982 3203832510.3389/fpsyt.2019.00982PMC6987424. doi:10.3389/fpsyt.2019.00982.

24.

Reichenberg A , Mollon J. Challenges and opportunities in studies of cognition in the prodrome to psychosis: No detail is too small. JAMA Psychiatry 2016;73:1249–502780615410.1001/jamapsychiatry.2016.2655.

25. See note 24, Reichenberg, Mollon 2016, at 1249–50.

26.

Rutter M. Biomarkers: Potential and challenges. In Singh I , Sinnott-Armstrong W , Savulescu J , eds. Bioprediction, Biomarkers, and Bad Behavior: Scientific, Legal and Ethical Challenges. Oxford: Oxford University Press; 2013:188–205, at 201 .

27.

Wexler B. Brain and Culture: Neurobiology, Ideology, and Social Change. Cambridge, MA: MIT Press; 2008, at 5 ff.

28. See note 16, Castle, Buckley 2015, at 76.

29.

Lee S , Ripke S , Neale B , Faraone S , Purcell S , Perlis R , et al. Genetic relationship between five psychiatric disorders estimated from genome-wide SNPs. Nature Genetics 2013;45:984–94, at 984 2393382110.1038/ng.2711PMC3800159.

30. See note 29, Lee et al. 2013, at 984.

31. See note 1, McIntosh et al. 2019.

32. See note 24, Reichenberg, Mollon 2016, at 189.

33.

Baum M. The Neuroethics of Biomarkers: What the Development of Bioprediction Means for Moral Responsibility, Justice and the Nature of Mental Disorder. Oxford: Oxford University Press; 2016, at chap. 2.

34.

Schmaal L , van Harmelen A-L , Chatzi V , Lippard E , Toenders Y , Averill L , et al. Imaging suicidal thoughts and behaviors: A comprehensive review of 2 decades of neuroimaging studies. Molecular Psychiatry 2020;25:408–27.3178775710.1038/s41380-019-0587-xPMC6974434

35.

Abbar M , Demattei C , El-Hage W , Llorca P-M , Samalin L , Demaricourt P , et al. Ketamine for the acute treatment of severe suicidal ideation: Double blind, randomised placebo-controlled trial. BMJ 2022;376:e06719410.1136/bmj-2021-067194PMC880846435110300. doi:10.1136/bmj.2021.067194.

36.

Hirschtritt M , Kroenke K. S creening for depression. Journal of the American Medical Association 2017;318:745–6; Thombs B, Markham S, Rice D, Ziegelstein, R. Does depression screening in primary care improve mental health outcomes? BMJ 2021;374:n16613428190810.1136/bmj.n1661. 10.1136.bmj.b1661.

37.

Cuijpers P , Beekman A , Reynolds C. Preventing depression: A global priority. Journal of the American Medical Association 2012;307:1033–4, at 1033 2241609710.1001/jama.2012.271PMC3397158.

38.

Singh I , Rose N. Biomarkers in psychiatry. Nature 2009;460:202–7, at 2041958776110.1038/460202a.

39.

Feinberg J. The child’s right to an open future. In Feinberg J , ed. Freedom and Fulfillment: Philosophical Essays. Princeton, NJ: Princeton University Press; 1992:76–97 .

40. See note 35, Abbar et al. 2022.

41. See note 2, Engert et al. 2020.

42.

Kane J , Robinson D , Schooler N , Mueser K , Penn D , Rosenheck R , et al. Comprehensive versus usual community care for first-episode psychosis: 2-year outcomes from the NIHM RAISE early treatment program. American Journal of Psychiatry 2016;173:362–722648117410.1176/appi.ajp.2015.15050632PMC4981493.

43.

Scarpazza C , Ha M , Baecker L , Garcia-Dias R , Pinaya W , Vieira S , et al. Translating research findings into clinical practice: A systematic review of neuroimaging-based clinical tools for brain disorders. Translational Psychiatry 2020;10:107 3231300610.1038/s41398-020-0798-6PMC7170931. doi:10.1038/s41398-020-0798-6.

